# Concurrent validity of different functional and neuroproteomic pain assessment methods in the rat osteoarthritis monosodium iodoacetate (MIA) model

**DOI:** 10.1186/s13075-016-1047-5

**Published:** 2016-06-23

**Authors:** Colombe Otis, Julie Gervais, Martin Guillot, Julie-Anne Gervais, Dominique Gauvin, Catherine Péthel, Simon Authier, Marc-André Dansereau, Philippe Sarret, Johanne Martel-Pelletier, Jean-Pierre Pelletier, Francis Beaudry, Eric Troncy

**Affiliations:** Groupe de Recherche en Pharmacologie Animale du Québec (GREPAQ), Department of Biomedical Sciences, Faculty of veterinary medicine, Université de Montréal, 1500 des Vétérinaires Street, P.O. Box 5000, St-Hyacinthe, Quebec J2S 7C6 Canada; Osteoarthritis Research Unit, Research Center Hospital of Montreal University (CRCHUM), Montreal, Quebec Canada; Department of Physiology and Biophysics, Faculty of Medicine and Health Sciences, Université de Sherbrooke, Sherbrooke, Quebec Canada; CiToxLAB North America Inc., Laval, Quebec Canada

**Keywords:** Animal preclinical model, Osteoarthritis, Monosodium iodoacetate, Methods, Validation, Acclimatization, Pain metrology, Neuropeptide

## Abstract

**Background:**

Lack of validity in osteoarthritis pain models and assessment methods is suspected. Our goal was to 1) assess the repeatability and reproducibility of measurement and the influence of environment, and acclimatization, to different pain assessment outcomes in normal rats, and 2) test the concurrent validity of the most reliable methods in relation to the expression of different spinal neuropeptides in a chemical model of osteoarthritic pain.

**Methods:**

Repeatability and inter-rater reliability of reflexive nociceptive mechanical thresholds, spontaneous static weight-bearing, treadmill, rotarod, and operant place escape/avoidance paradigm (PEAP) were assessed by the intraclass correlation coefficient (ICC). The most reliable acclimatization protocol was determined by comparing coefficients of variation. In a pilot comparative study, the sensitivity and responsiveness to treatment of the most reliable methods were tested in the monosodium iodoacetate (MIA) model over 21 days. Two MIA (2 mg) groups (including one lidocaine treatment group) and one sham group (0.9 % saline) received an intra-articular (50 μL) injection.

**Results:**

No effect of environment (observer, inverted circadian cycle, or exercise) was observed; all tested methods except mechanical sensitivity (ICC <0.3), offered good repeatability (ICC ≥0.7). The most reliable acclimatization protocol included five assessments over two weeks. MIA-related osteoarthritic change in pain was demonstrated with static weight-bearing, punctate tactile allodynia evaluation, treadmill exercise and operant PEAP, the latter being the most responsive to analgesic intra-articular lidocaine. Substance P and calcitonin gene-related peptide were higher in MIA groups compared to naive (adjusted *P* (adj-*P*) = 0.016) or sham-treated (adj-*P* = 0.029) rats. Repeated post-MIA lidocaine injection resulted in 34 times lower downregulation for spinal substance P compared to MIA alone (adj-*P* = 0.029), with a concomitant increase of 17 % in time spent on the PEAP dark side (indicative of increased comfort).

**Conclusion:**

This study of normal rats and rats with pain established the most reliable and sensitive pain assessment methods and an optimized acclimatization protocol. Operant PEAP testing was more responsive to lidocaine analgesia than other tests used, while neuropeptide spinal concentration is an objective quantification method attractive to support and validate different centralized pain functional assessment methods.

## Background

Osteoarthritis (OA), the most common of all arthropathies in our aging population, is a leading cause of disability and represents a large (and growing) worldwide socio-economic cost [1]. It affects approximately 30 million adults in the USA [2], and this number is expected to double by 2020 [3], with longer life expectancy and the increasing incidence of obesity, two major risk factors for the disease. Despite critical importance in drug development, translation of OA therapies focusing either on structure (disease-modifying OA drugs) or pain (symptom-modifying OA drugs) from the bench to bedside has slowed [1, 4, 5]. Differences between preclinical OA models and the disease evaluated in clinical trials contribute to this failure. Rising criticism is noted over the classic translational research, which has failed to predict the efficacy of chronic pain treatments [6–9]. Most critics have targeted the poor validity and clinical relevance of experimental pain models using laboratory animals [10, 11]. It has also been hypothesized that current animal models are too reliant on evoked (reflexive) withdrawal responses and that development of meaningful assessment tools allowing, for instance, the measurement of continuous spontaneous pain, might help to translate experimental data to clinical practice [6, 12].

Naturally occurring OA models are recognized to present pathophysiological changes closest to clinical OA, particularly in large animals [13], but also entail experimental disadvantages (long period to onset, and variability of disease development). In contrast, chemical models cause the most rapidly progressing OA, requiring less invasive procedures and enabling standardization (with increased sample homogeneity). The monosodium iodoacetate (MIA) chemical OA model as described 25 years ago induces cartilage degeneration by disruption of chondrocyte metabolism (i.e., breaking down the cellular aerobic glycolysis). In rats, the MIA model is well-established and resembles the histological and pain-related characteristics of human degenerative OA [14–29]. Owing to the extensive description of the pain response in rats, the MIA OA model was proposed as a standard OA model for pain assessment [23, 30].

The quality of pain assessment methodologies is a cornerstone of preclinical studies targeting new analgesics [7, 10–12, 16, 31, 32]. Three different categories of pain expression can be evaluated in rats: reflexive measures, spontaneous measures, and operant responses [8, 31]. First, reflexive measures using stimulus-evoked responses are commonly used in rats to assess potential hyperalgesia and allodynia, recognized as a clinical expression of the neuropathic pain (nociceptive sensitization) component [8, 33–35]. These measures are generated by exposure to thermal, mechanical, or electrical stimulus, involving mainly spinal-level pain processing, and are also increasingly present in human quantitative sensory testing characterization of pain [36–38]. Second, spontaneous measures can be useful to quantify pain and/or wellbeing [8]. For example, kinetic (static or dynamic weight distribution) [14] or kinematic [39] (ambulation evaluation or characterization) measurement, and spontaneous activity [24] can indirectly assess quality of life in OA models. Pain-induced behaviors (scratching/licking/biting, hypophagia, vocalization, etc.) should also be considered in this category [37]. Finally, operant responses have been more recently introduced to characterize pain in animal models [8, 34, 40–44]. Operant testing is opposite to reflexive response testing as it allows the quantification of behavioral responses at higher levels of the brain, reproducing multiple dimensions of pain, including affective and cognitive changes and not only sensory-discriminative perception [42, 43, 45, 46]. This type of measure allows the observer to evaluate the aversive component of pain as operant tests give the animal an opportunity to avoid the painful condition [33, 40–43, 47–49].

In patients OA of the knee joint, pain is a combination of inflammatory, immune and neurogenic components participating in the hypersensitivity syndrome. Central sensitization mechanisms [50] include various biochemical processes such as increased spinal release of neurotransmitters and neuromodulators, and increased excitability of postsynaptic neurons. In an OA [51] and arthritis [52] rat model, higher levels of neuropeptides, such as substance P (SP) and calcitonin gene-related peptide (CGRP) have been found in the spinal cord. Thus, nervous system modulation seems to play a critical role in the development of the disease [53]. The contribution of these spinal neuromediators to neurogenic inflammation-mediated chronic pain in OA, and concomitant changes in functional pain assessment methods has not been fully established.

With such a variety of methods for pain and analgesic response assessment, it is difficult to opt for the method(s) most adapted to specific conditions. The current study undertook to establish, the reliability of a panel of pain assessment methods (including reflexive, spontaneous, and operant testing) in normal rats, and the influence of environmental conditions, including acclimatization and experimental conditions of manipulation (observer, inverted circadian cycle, and exercise). The most reliable methods were then used to characterize OA pain in the well-established chemical MIA model in rats, while conducting concurrent validation of pain assessment methods in relation to the expression of different spinal neuropeptides and their responsiveness to treatment with intra-articular lidocaine.

## Methods

### Ethics statement

During the study, care and use of animals were subject to and approved by the Comité d’Éthique de l’Utilisation des Animaux of Université de Montréal (#Rech-1495) and conducted in accordance with principles outlined in the current Guide to the Care and Use of Experimental Animals published by the Canadian Council on Animal Care and the Guide for the Care and Use of Laboratory Animals published by the US National Institutes of Health.

### Animals

The present study was conducted on female (n = 63; excluding spares) Sprague-Dawley rats (Charles River Laboratories, Saint-Constant, QC, Canada) ranging from 225–300 g in weight at the beginning of experiment. The animals were housed under regular laboratory conditions and maintained under a light-dark cycle with food and water provided *ad libitum*. Body weight was obtained weekly. At the end of each experiment, the animals were returned to their housing colony.

### Phase 1: reliability of pain assessment methods in normal rats

#### Experimental design

Phase 1 included a total of 39 normal rats distributed into 8 groups. First, the repeatability of measurements was tested for the influence of environment, including observer, inverted circadian cycle (activity during the day), and exercise (two groups of five animals in crossover). Additionally, repeatability over an extended period was tested again for static weight-bearing (SWB; one group of four animals also tested for exercise effect), and for tactile sensitivity and place escape/avoidance paradigm (PEAP) operant test (one group of five animals). Second, using the most reliable methods only, the influence of four acclimatization protocols (four groups of five animals) was tested to determine the most effective approach to obtain predictable data with low variability. Different pain assessment methods were selected to include reflexive, spontaneous behavior and operant measures.

#### Influence of environment

First, rats (n = 10) were randomly distributed into two groups of five. Animals were acclimated to the test apparatus on two occasions at day -3 and -1 before starting the experimentation. In a crossover design, the animals were subsequently assessed for three repeated days during light (1000–1400 h) and dark (2000–2400 h) cycles to test the influence of inverted circadian cycles. Both cycles were separated by a 3-day washout period without assessment. Dark cycle evaluations were performed under low-intensity red light. Animals were tested on each of six assessment days by two observers, with the following methods in this order of evaluation: mechanical and tactile sensitivity, SWB, treadmill exercise, mechanical and tactile sensitivity, SWB, PEAP operant test (without nociceptive stimulation) and rotarod acceptance. The mechanical and tactile nociceptive thresholds and SWB evaluation were performed before and after the treadmill exercise to verify the influence of exercise on these three pain assessment methods. Finally, PEAP and rotarod were performed at the end of the evaluation schedule to ensure respectively, that the length of the test, and possible falling from the test device would not impair the other outcomes. To test inter-rater reliability, two female observers were selected for their different levels of experience in laboratory animals (one intermediate, one with advanced expertise).

Second, in complementary studies about test repeatability (after acclimatization on two occasions, at day -3 and -1), SWB was specifically retested over 15 days with two SWB assessments separated by a treadmill session (then testing again the potential effect of exercise on SWB) in a group of four rats. This evaluation was done daily, from days 1 to 5, then on days 8 and 15.

Finally, the repeatability of measurements of tactile sensitivity and PEAP (with nociceptive stimulation) was tested in a group of five rats over 25 days. These evaluations were done daily, from days 1 to 15, and repeated on days 18 and 25.

#### Influence of acclimatization protocols

In order to determine the most efficient acclimatization protocol associated with the most repeatable data (previously obtained), the next experiment was conducted in a total of 20 animals (four groups of 5 animals). Briefly, over 2 weeks, different acclimatization protocols were tested and included 8 (days -14, -13, -12, -11, -10, -8, -6, and -1), 6 (days -14, -8, -7, -6, -5, and -1), 5 (days -14, -7, -5, -3, and -1) or 4 (days -14, -8, -6, and -1) days of evaluation. The order of assessment was SWB, tactile and mechanical sensitivity, PEAP with nociceptive stimulation, and treadmill. The schedule of pain evaluation methods was determined to obtain nociceptive threshold values before placing the animal on the operant testing device where many paw stimulations were elicited (see subsequent description).

#### Pain assessment methods

##### Mechanical sensitivity

Mechanical sensitivity was assessed by measuring the paw withdrawal threshold (PWT) to an increasing pressure stimulus placed on the dorsal surface of the hind paw using an algorimeter (Randall Selitto test Paw Pressure Meter®, IITC Life Science Inc., Woodland Hills, CA, USA), employing a wedge-shaped probe (1.75 mm^2^ of surface) and a cutoff value set at 250 g. The animals were placed in a sling apparatus (Lomir Biomedical Inc., Notre-Dame-de-l’Île-Perrot, QC, Canada). The probe was applied once on the dorsal surface at a steadily increasing pressure. The PWT was determined when the animal removed the paw from the apparatus, and the required pressure was recorded. Withdrawal thresholds were measured on the right and left hind paws. The data were expressed as PWT in grams.

##### Tactile sensitivity

First, the animal was placed inside an elevated metal grid cage to allow just enough space for the rat to move while being restricted. After the rat exploration session during the first 2 minutes, tactile sensitivity was assessed using an Electronic von Frey Anesthesiometer® (IITC Life Science Inc., Woodland Hills, CA, USA) applied to the plantar surface of the hind paws and by measuring the PWT to von Frey ascending mechanical stimuli. Gradually increasing pressure was applied with a mechanical von Frey polypropylene probe (0.7 mm^2^, Rigid Tip®, IITC Life Science Inc., Woodland Hills, CA, USA) fitted to a handheld force transducer. The rigid tip was placed perpendicularly into the mid-plantar surface of the paw. The stimulus was continued until the hind paw was withdrawn or elevated such that the force leveled off. Actions such as vocalization, agitation, jumping, and avoidance were considered indicative of the PWT. Voluntary movements associated with locomotion were not considered to be a withdrawal response. The peak of force in grams was recorded with a cutoff value at 100 g. For each animal, triplicates of each hind paw were taken with a 60-s interval between each stimulus.

##### Static weight bearing

The weight distribution through the right and left knee was assessed using an Incapacitance Meter® (IITC Life Science Inc., Woodland Hills, CA, USA) to measure SWB distribution in the two hind limbs. The force exerted by each hind limb was measured and analyzed in grams, but reported in percentage of total body weight (%BW) to normalize the data. Rats were allowed to acclimate to the testing apparatus and when stationary, readings were taken over a 3-s period. Triplicates were taken simultaneously for each limb at each time point.

##### Treadmill exercise

All rats underwent forced training over a 20-minute period at constant treadmill speed (11 m/minute) (IITC Life Science Inc., Woodland Hills, CA, USA). To force the animal to exercise on the treadmill belt, each lane was equipped with an independent shocker grid. The intensity of the shocker grid was kept at the minimum required to keep the animal on the exercise belt. The treadmill number of total crossings (TNTC) was recorded over the whole period, but also reported in blocks of 5 minutes, to potentially detect a within-time change in activity. A total crossing was considered completed when the animal crossed the entire length of the lane. The TNTC was used as an indicator of exercise and/or performance. When the rats were running continuously on the belt, they were exposed to maximal intensity exercise, as they were not pausing, causing them to cross the entire length of the motorized lane.

##### Operant testing

The PEAP was used as operant testing apparatus [33, 40, 49]. Rats were placed into test cage apparatus that was painted half white and half black. Neither side was illuminated with additional light. With the cage on an elevated metal grid, the observer, located below, determined the preferential location of the rat. The 20-minute observation period began after 2 minutes of acclimatization/exploration to the test environment on each occasion.

##### Operant testing without nociceptive stimulation

The percentage of time spent on the black or white side of the test apparatus was calculated from observation of the preferential location every 15 s.

##### Operant testing with nociceptive stimulation

If the rat was on the black side of the test apparatus, the plantar surface of the right (ipsilateral to possible MIA intra-articular injection) hind paw (RHP) was stimulated with a thin wire (60 g) every 15 s, to prompt withdrawal of the limb. When the rat was on the white side of the cage, a similar mechanical stimulation was applied, but to the plantar surface of the left (contralateral) hind paw (LHP). The percentage of time in the black and white side of the test apparatus was calculated from observation of the preferential location every 15 s. The calculations were sequenced by successive blocks (n = 4) of 5-minute periods. Moreover, the total number of crossings from the white to the dark side was noted to detect any decrease in activity. If a rat remained in the crossing tunnel, it would be stimulated to advance and complete its crossing.

##### Rotarod

Using a Rotamex 4/8® (Columbus Instruments Inc., Columbus, OH, USA) with a previously published protocol [29], the rats were exposed to an acceleration speed of 5 to 16 rpm, over 60 s, before being maintained at this speed, while the time before falling was monitored with a cutoff time of 3 minutes.

### Phase 2: concurrent validity with the MIA model

#### Experimental design

In the second phase, a pilot study (n = 24 rats) was conducted to test the concurrent validity of different functional and neuropeptide pain assessment methods in the MIA rat OA model. A single intra-articular injection of MIA was performed in the right knee of 16 animals distributed among two groups (n = 8 each). An additional sham group (n = 8) received a single intra-articular injection (50 μL) of 0.9 % NaCl. For the purpose of the study, one of the two MIA groups also received a punctual lidocaine (L) injection (MIA-L group) in the right knee on days 7, 14 and 21. At the end of the 21 days of the experimentation, all animals were euthanized with an overdose of isoflurane and a sacrifice by transection of the cervical spine before spinal cord collection.

#### Acclimatization period and baseline assessment

The study began with an acclimatization period for the selected optimal outcomes (SWB, tactile sensitivity, PEAP, rotarod, and treadmill), according to the optimal acclimatization protocol of five occurrences (days -14, -7, -5, -3, and -1) obtained in phase 1. Because of pain induction in phase 2, tactile sensitivity could be considered as punctate tactile allodynia evaluation (PTAE). Baseline values were acquired at day -1 in this order of evaluation, following the above-described testing procedures: SWB, PTAE, and PEAP with nociceptive stimulation, rotarod and treadmill, with intra-articular injection of MIA at day 0 in the right knee.

#### Intra-articular injection

On day 0, fasted (3–6 h) rats from all groups were premedicated with buprenorphine hydrochloride (0.02 mg/kg IM; Buprenex® injectable, Reckitt Benckiser Inc., Mississauga, ON, Canada) and mask-anesthetized with a 2 % isoflurane–O_2_ mixture. After surgical preparation, a single intra-articular injection of 2 mg MIA (monosodium iodoacetate, BioUltra®, ≥98 %, Sigma-Aldrich Canada Co., no. I9148-5G, Oakville, ON, Canada) dissolved in isotonic saline, or saline 0.9 % (both 50 μL volume) was administered through the infrapatellar ligament of the right knee, using a 26-gauge, 0.5-inch needle mounted on a 0.5-mL syringe. On days 7, 14, and 21 post-MIA injection, 25 minutes before functional assessment, rats from the MIA-L group were again similarly anesthetized with a single intra-articular injection of lidocaine through the infrapatellar ligament of the right knee. Lidocaine Neat® (2 %, Zoetis Canada, Kirkland, QC, Canada) was injected at a volume of 50 μL using a 26-gauge, 0.5-inch needle mounted on a 0.5-mL syringe.

#### Post-injection evaluation

The assessments were performed according to the specific schedules of the different groups on days 3, 7, 14, and 21 post injection, and conducted as described for phase 1. For the MIA-L group on days 7, 14, and 21, the evaluation started 25 minutes after the animals recovered from anesthesia. The evaluation sequence was as follows: SWB (%BW), PTAE (grams), PEAP (percentage of time spent on the dark side), rotarod (seconds) and treadmill (TNTC). The schedule of evaluation was designed to obtain the SWB at rest and the PTAE data before the operant testing evaluation, as this test elicits many PWT stimulations.

#### Proteomic analysis

##### Reagents and solutions

Acetic anhydride 99.5 % (Ac_2_O) and ammonium bicarbonate (NH_4_HCO_3_) were obtained from Sigma-Aldrich Inc. (St Louis, MO, USA). SP and CGRP were purchased from Phoenix Pharmaceuticals Inc. (Belmont, CA, USA). Acetonitrile was purchased from Thermo Fisher Scientific Inc. (NJ, USA), and trifluoroacetic acid, formic acid and ammonium hydroxide 28.0–30.0 % (NH4OH) were purchased from J.T. Baker® (Phillipsburg, NJ, USA). Standard solutions were prepared as previously performed [54].

##### Instrumentation

The tandem mass spectrometry coupled to high-performance liquid chromatography (HPLC-MS/MS) system comprises a Thermo Surveyor autosampler, a Thermo Surveyor MS pump and a Thermo LCQ Advantage Ion Trap Mass Spectrometer (Thermo Fisher Scientific Inc., San Jose, CA, USA). Data were acquired and analyzed with Xcalibur^TM^ 1.4 (Thermo Fisher Scientific Inc., San Jose, CA, USA), and regression analysis were performed with PRISM® (version 5.0d) (GraphPad software Inc., La Jolla, CA, USA) using the nonlinear curve fitting module with an estimation of the goodness of fit. The calibration lines were constructed from the peak-area ratios of targeted neuropeptides (SP or CGRP) and the acetylated SP analog internal standard.

##### Bioanalytical methods

Acetylated SP was used as the internal standard. The reaction was performed as previously described [54] and the analytical method used was also based on a previously published method [55]. The internal standard solution was tested by HPLC-MS/MS in multiple reactions monitoring (MRM) mode and no residual SP were detected.

##### Spinal cord sample preparation

At the end of the 21 days of experimentation, the entire spinal cord tissue of rats (n = 24) was rapidly collected by a flush of saline within the lumbar spinal canal following deep anesthesia with isoflurane and sacrifice by transection of the cervical spine. Samples were snap-frozen in liquid nitrogen and stored at –80 °C pending analysis. Each spinal cord was weighed accurately and homogenized using a tissue tear or following the addition of phosphate-buffered saline solution (PBS) 0.01 M at a ratio of 1:5 (v/v) and protease inhibitor cocktail (Sigma-Aldrich Inc., Oakville, ON, Canada, number PP8340) at the same ratio. The samples were sonicated and the homogenate was mixed with acetonitrile at a ratio of 1:1 (v/v) to remove larger proteins. The samples were vortexed and centrifuged for 10 minutes (×12,000 g) and the supernatant was transferred into an injection vial then spiked with the internal standard solution at a ratio of 1:1 (v/v). The spinal cords from a naive group (n = 5) in phase 1 were also collected to obtain a baseline value from normal rats to normalize values obtained from the MIA, MIA-L, and sham groups.

### Statistical analysis

All statistical analyses were performed two-sided with an alpha value set at 0.05 (phase 1) or 0.10 (phase 2) using a statistical software program (SAS system for Windows, version 9.2, Cary, NC, USA). The alpha value for phase 2 was set at 0.1 because this phase was an exploratory study. In a pilot study, it is acceptable to set a higher alpha value when the study has the hopes of finding an effect that could lead to a promising scientific discovery [56] in order to increase the power (consequently decreasing the risk of type II error), but increasing the chances of type I error (i.e.*,* saying there is a difference when there is not). To be consistent with the statistical rules of correction for multiple comparisons, phase 2 results were presented as adjusted *p* values (adj-*P*) because the values obtained in the statistical report need to be multiplied by the total number of comparisons. The normality of the outcomes was verified using the Shapiro-Wilk test and the homogeneity of variance was assessed using the absolute values of the residuals of the mixed model, when appropriate.

#### Phase 1: reliability of pain assessment methods in normal rats

For mechanical nociceptive thresholds and SWB, the effect of the circadian cycle was assessed using the paired *t* test adapted for a crossover design. Moreover, the effect of covariates of interest, namely observer, exercise, limb (when both left and right limbs were tested), or trials (when replicates were conducted), was assessed using a general linear model. Generalized linear mixed model analyses for repeated measures were conducted to test the effect of groups on TNTC and rotarod (lognormal distribution), and PEAP (Poisson distribution). Models accounted for baseline measurements using the baseline as covariates. This enabled assessment of the effect of the procedure over time using each subject as its own control. For each model, the best structure of the covariance model was assessed using information criteria that measure the relative fit of competing covariance models. When comparing the 5-minute periods, the Bonferroni adjustment was applied (initial alpha value divided by 4).

Outcome repeatability (test-retest reliability) was assessed by computing the intraclass correlation coefficient (ICC). The ICC is a measure of the proportion of variance that is attributable to objects of measurement. Quantifying the test-retest reliability, the closer the ICC is to 1.0, the higher the reliability and the lower the error variance [57]. A ratio of 0.3–0.4 indicates fair agreement, 0.5–0.6 moderate agreement, 0.7–0.8 strong agreement, and >0.8 almost perfect agreement. Moreover, the coefficient of variation (CV), as a normalized measure of dispersion of the distribution, was used to test the effect of the proposed acclimatization protocols. The CV for each variable was calculated at day -14 (initial assessment), and the variation in CV was assessed at the end of each acclimatization protocol as the CV ratio of day -1 (final assessment) to day -14. At the initial assessment (day -14), the CV interpretation was as follows: <10 % indicated almost perfect dispersion, 11–25 % light dispersion, and 26–40 % fair dispersion. The day -1/day -14 CV ratio indicated improvement (decrease in variability) related to the acclimatization protocol if it was <1, and deterioration (increase in variability) if >1.

#### Phase 2: concurrent validity with the MIA model

The SWB and PTAE data were expressed as the average obtained from the three trials on the RHP. Data were then analyzed using linear mixed models (SWB and PTAE) or generalized linear mixed models for repeated measures. Treatment groups and day were considered as fixed effects and animals in groups as random effects. Models accounted for baseline measurement using the baseline as a covariate. For each model, the best structure of the covariance model was assessed using a graphical method (plots of covariance versus lag in time between pairs of observations compared to different covariance models), and using information criteria that measure the relative fit of competing covariance models. When multiple comparisons were carried out, the Tukey-Kramer adjustment was used to obtain adj-*P* values. Neuropeptide data were analyzed using the unpaired exact Wilcoxon test with an alpha value set at 0.10 following non-parametric Kruskal-Wallis one-way analysis of variance.

## Results

### Phase 1: reliability of pain assessment methods in normal rats

#### Data variability and influence of environment

The repeatability of measurements made with different assessment methods was tested in normal rats, and the influence of environment, including observer, inverted circadian cycle (activity during the day), exercise, limb, and trial, was assessed on testing.

##### Mechanical sensitivity

We did not find any effect of observer, circadian cycle, exercise, or limb in the PWT measured with the Randall Selitto test Paw Pressure Meter®. However, the data obtained with this test were highly variable among individuals and not repeatable (ICC <0.3).

##### Tactile sensitivity

The PWT measured with the Electronic von Frey Anesthesiometer® in normal rats gave average values of 40–80 g in both hind limbs. The observer, the circadian cycle, and exercise did not produce any effect on tactile sensitivity. No significant difference between the right and left hind limbs, or trial effect (in the triplicates) was observed. However, the data were markedly variable over the whole period (6 days in total) with an ICC for both hind limbs <0.5. Following repetition of the experiment in five rats over 25 days, the ICC improved after excluding the first 2 weeks of daily evaluation. More precisely, the ICC for days 15, 18, and 25 was >0.8 for both hind limbs (Table 1).

**Table 1:** Test-retest reliability of the tactile sensitivity evaluation

	LHP	RHP
ICC over the whole period	0.78	0.26
ICC for days 7, 15, 18, and 25	0.79	0.27
ICC for days 15, 18, and 25	0.84	0.81

A group of normal rats (n = 5) was tested with the Electronic von Frey Anesthesiometer® daily from days 1 to 15 and then on days 18 and 25. The intraclass correlation coefficients (ICCs) for values calculated for the entire evaluation period were compared to values calculated after exclusion of the first week and the first two weeks of assessment. *LHP* left hind paw, *RHP* right hind paw

##### Static weight bearing

In normal animals, the Incapacitance Meter® apparatus measured average values of weight distributed over each hind limb between 35 and 38 % BW. The observer, the circadian cycle, and exercise did not produce any effect on SWB. No significant difference between the right and left hind limbs, or trial effect (in the triplicates) was observed. When analyzing the last two days of assessment (in comparison to the whole period of 6 days), the ICC improved (Table 2), and this was particularly evident for the SWB ICC after exercise (ICC >0.7 in both hind limbs for the last 2 days of assessment). This suggests that the treadmill exercise slightly decreased the inter-individual variability in SWB measurement. Finally, following repetition of evaluation over 15 days with in four rats, the ICC improved after excluding the first week of daily evaluation (days 1 to 5), with a value ≥0.66 for days 8 and 15 in both hind limbs (Table 2).

**Table 2:** Test-retest reliability of the static weight bearing

	Before exercise		After exercise	
	LHP	RHP	LHP	RHP
ICC over the whole period	0.00	0.00	0.00	0.23
ICC for days 8 and 15	0.67	0.66	0.66	0.76

A group of normal rats (n = 4) was tested for static weight bearing before and after treadmill exercise daily from days 1 to 5 and then on days 8 and 15. The intraclass correlation coefficients (ICCs) calculated for the entire evaluation period were compared to ICCs calculated after exclusion of the first week of assessment. *LHP* left hind paw, *RHP* right hind paw

##### Treadmill

**Fig. 1:**
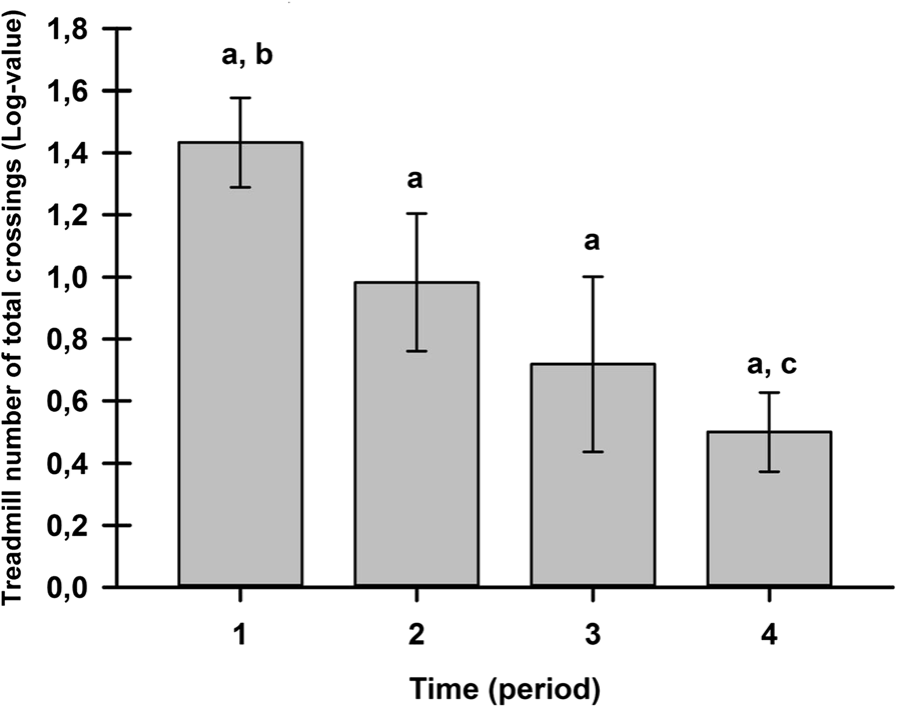
Treadmill exercise repeatability (least squares mean ± standard error of the mean). A group of four animals was tested on the treadmill, recording the number of total crossings over 20 minutes (period 1 = 0–5 minutes, period 2 = 5–10 minutes, period 3 = 10–15 minutes, and period 4 = 15–20 minutes), daily from days 1 to 5, and then on days 8 and 15. The treadmill numbers of total crossings were transformed to fit a lognormal distribution. ^a,b,c^ Statistically significantly different inter-period statistical differences (adjusted *P* value = 0.002

The treadmill exercise sessions were generally well accepted by female Sprague-Dawley rats (84 % acceptability). Neither the observer, nor the circadian cycle produced any effect on the TNTC. The TNTC was extremely repeatable with an ICC of 0.84. A period effect was demonstrated (*P* = 0.003) in the 15-day study in four rats (Fig. 1). Post hoc analysis showed that the initial and final 5-minute periods were different for TNTC (*P* = 0.0002), whereas both intermediate 5-minute periods (numbers 2 and 3) were highly repeatable with an ICC of 0.73 and 0.92, respectively.

##### Place escape/avoidance paradigm

**Fig. 2:**
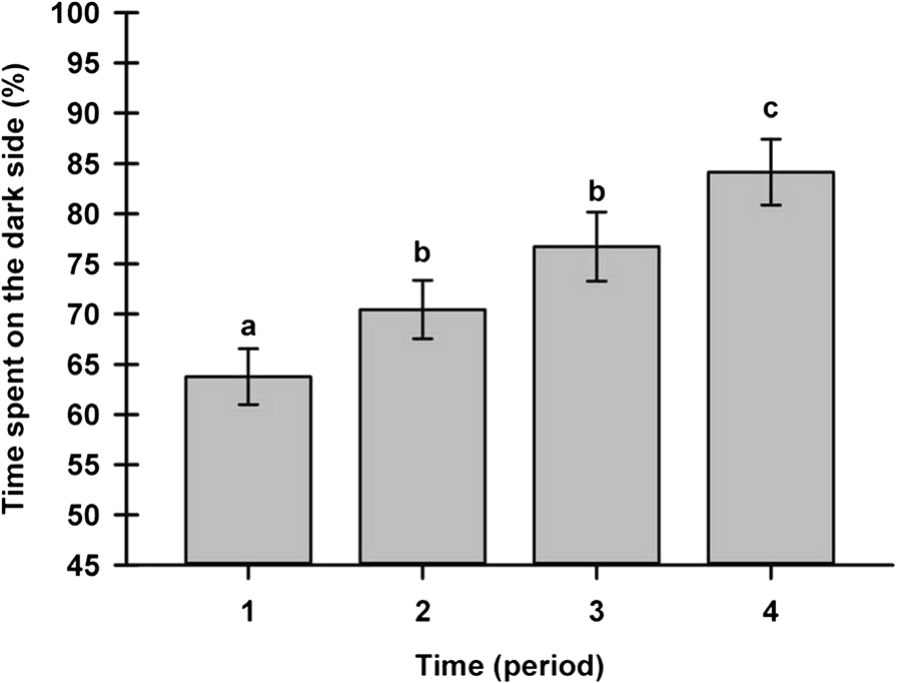
Place escape/avoidance paradigm operant test repeatability (least squares mean ± standard error of the mean). A group of five animals underwent the place escape/avoidance parading operant test (percentage of the time spent on the dark side) over a 20-minute (period 1 = 0–5 minutes, period 2 = 5–10 minutes, period 3 = 10–15 minutes, and period 4 = 15–20 minutes) daily from days 1 to 15, and then on days 18 and 25. ^a,b,c^ Significant inter-period differences (adjusted *P* value ≤0.0216)

The first experiment with operant testing was done without nociceptive stimulus. Neither the observer, nor the circadian cycle produced any effect on the preferential localization. The localization was highly repeatable among animals, with an ICC of 0.90, where the rats spent 91 % of their time on the black side. A period effect was demonstrated (*P* < 0.0001) in the 25-day study of five rats (Fig. 2). The PEAP assessment with nociceptive stimulation once again demonstrated robust repeatability, with an ICC of 0.83, and rats spent 81 % of their time on the black side. The post hoc analysis showed that the intermediate 5-minute periods (numbers 2 and 3) were similar in the percentage of time, and were highly repeatable and different (*P* ≤ 0.0216) from the initial and final 5-minute periods.

##### Rotarod

Neither the observer, nor the circadian cycle produced any effect on the performance time in the rotarod, and this performance time was highly repeatable with an ICC of 0.92.

#### Influence of acclimatization protocols and comparison of assessment method variability

When looking at the different assessment methods for the initial day of acclimatization (day -14), inter-individual variability (CV) appeared lower for the SWB, followed by PEAP, tactile sensitivity, and treadmill (TNTC) evaluation, with mechanical sensitivity last (Table 3). The variation in CV at day -1, normalized to day -14, as tested by the day -1/day -14 CV ratio, was compared between acclimatization protocols for the different pain assessment methods (Table 3). The most intensive protocol with the highest number of acclimatization procedures (n = 8) presented the lowest variability between days -1 and -14, similar to the protocol with 6 or 5 days of acclimatization. The protocol with only 4 days of acclimatization yielded the highest variations in CV. The acclimatization protocol using five occurrences of exposition to different assessment methods appeared the most appropriate to limit variability in assessment.

**Table 3:** Coefficient of variation (CV) for each outcome and variation in CV between four protocols of acclimatization

	8 Days		6 Days		5 Days		4 Days	
Functional evaluation^a^	CV (D-14)	CV* ratio	CV (D-14)	CV* ratio	CV (D-14)	CV* ratio	CV (D-14)	CV* ratio
SWB	17.3	***0*** *.* ***72***	31.2	***0*** *.* ***30***	11.7	***0*** *.* ***62***	12.2	***0*** *.* ***67***
PEAP	23.8	1.01	24.1	1.07	21.3	0.97	22.8	1.11
TS	42.9	***0*** *.* ***85***	22.9	1.24	20.4	1.01	27.4	1.80
TNTC	36.9	1.10	106.5	***0*** *.* ***54***	31.2	0.91	38.0	1.92
MS	63.3	***0*** *.* ***74***	71.8	1.07	72.2	***0*** *.* ***71***	75.3	***0*** *.* ***53***

^a^The functional evaluation includes the values recorded for the right hind limb, when available (static weight bearing (SWB), tactile sensitivity (TS), mechanical sensitivity (MS)), or the response of the animal (place escape/avoidance paradigm (PEAP) and treadmill number of total crossings (TNTC)). *The CV values calculated on day -1 were normalized to the CV values on day -14 (D-14) to test the influence of the acclimatization protocol on the outcome measures. A day -1/day -14 CV ratio value <1 was indicative of improvement in variability and is presented in bold italics

### Phase 2: concurrent validity with the MIA model

The MIA injection successfully induced pain-related changes as assessed by SWB, PTAE, PEAP, and TNTC. However, the rotarod was not sensitive to MIA-induced pain, as all groups had similar (maximal) time of acceptance. In consequence, no further analysis was conducted with this testing modality. The sham injection was not totally neutral when compared to baseline values: while no effect was present for SWB or TNTC, the sham group had a transient decrease in PTAE (days 7 and 14) and PEAP (days 3 and 7). The response to lidocaine injection varied by assessment method: a clear analgesic effect was noted with PTAE (on days 7 and 14), and PEAP (on days 7, 14 and 21); a trend toward better performance was observed with TNTC, but no difference was observed with MIA for SWB. Neuropeptide spinal quantification permitted validation of the lidocaine treatment effect and the pain generated by MIA injection.

#### Static weight bearing

**Fig. 3:**
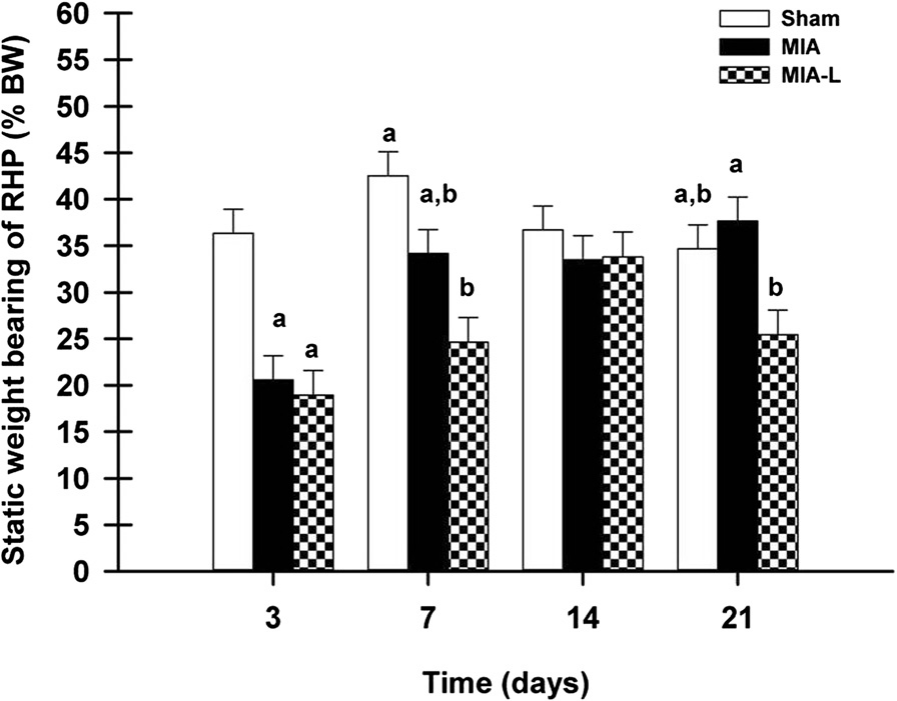
Static weight-bearing (SWB) evolution after induction of osteoarthritis (least squares mean ± standard error of the mean). On day 3, the monosodium iodoacetate (*MIA*) intra-articular injection in the right knee induced asymmetrical weight distribution in the rats injected with MIA (adjusted *P* value (adj-*P*) = 0.0024) and rats injected with MIA and punctual lidocaine (*MIA-L*) (adj-*P* = 0.0011) compared to rats injected with 0.9 % saline (sham). Subsequently, a statistically significant difference in right hind paw SWB was only observed between the sham and MIA-L groups at days 7 and 21. *%BW* percentage of body weight. ^a,b^Significant inter-group statistical differences

Analysis of SWB data demonstrated a group effect (*P* = 0.0005), a time effect (*P* < 0.0001) and a time *x* group effect (*P* = 0.005). In the MIA group, the nadir of weight force was observed on day 3 and was different from values recorded on days 7 (adj-*P* = 0.005), 14 (adj-*P* = 0.01) and 21 (adj-*P* = 0.001) (Fig. 3). No significant difference within time was observed for the sham group, whereas in the MIA-L group, day 3 RHP SWB (without lidocaine injection) was lower than on day 14 (adj-*P* = 0.001). Compared to the sham group, the RHP SWB decreased on day 3 in the MIA (adj-*P* = 0.002) and the MIA-L (adj-*P* = 0.001) groups. Subsequently, the RHP SWB in the MIA group returned to levels similar to those in the sham group, and at no time point of evaluation did the lidocaine injection provide any benefit.

#### Punctate tactile allodynia evaluation

**Fig. 4:**
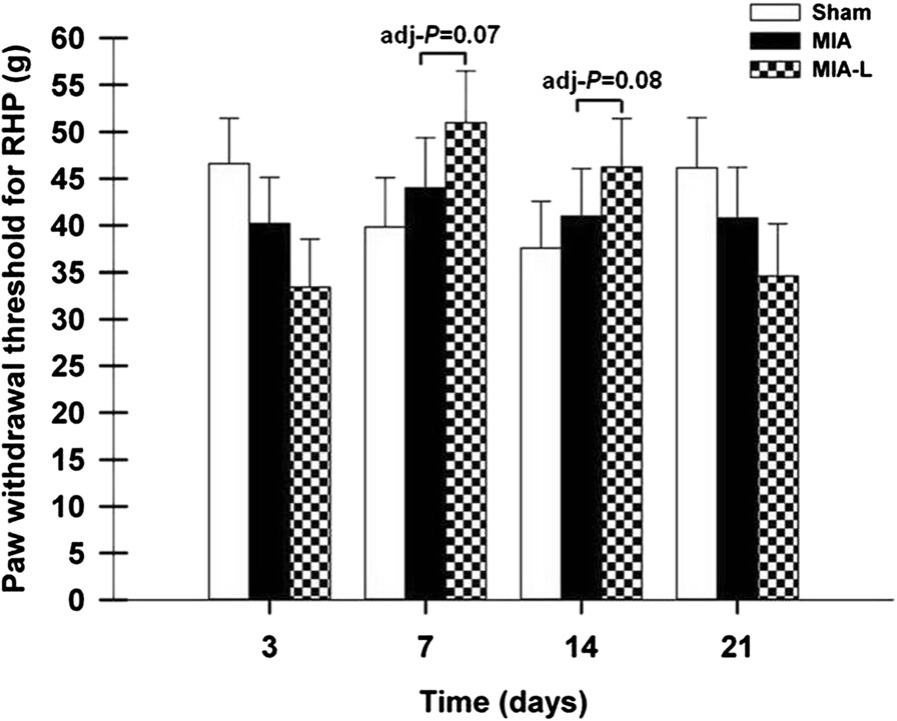
Right hind paw (*RHP*) withdrawal threshold evolution after induction of osteoarthritis (least squares mean ± standard error of the mean). On days 7 (adjusted *P* value (adj-*P*) = 0.07) and 14 (adj-*P* = 0.08), the RHP paw withdrawal threshold was increased for the rats injected with monosodium iodoacetate and punctual lidocaine (*MIA-L*) when compared with rats injected with MIA

Descriptive statistics for the RHP PTAE over the evaluation days are provided in Table 4. The PWT was lower after the MIA injection on days 3, 7, 14, and 21. This was also the case for the sham group on days 7 and 14. In the MIA-L group, the nadir in PWT was observed on day 3, whereas a significant increase was observed on days 7 and 14. There was a difference between MIA and MIA-L on days 7 (adj-*P* = 0.07) and 14 (adj-*P* = 0.08) (Fig. 4).

**Table 4:** Mean and standard deviation of the punctate tactile allodynia evaluation by experimental group over time

Experimental group	Days									
	-1		3		7		14		21	
	Mean	SD	Mean	SD	Mean	SD	Mean	SD	Mean	SD
MIA	53.8	11.2	39.2	13.1	43.1	13.7	40.0	10.0	39.8	8.5
MIA-L	70.4	16.8	35.0	13.8	52.6	12.7	47.8	14.3	36.2	17.8
Sham	56.3	16.9	49.0	14.8	39.2	16.6	37.0	17.6	45.6	17.0

Descriptive statistics of the punctate tactile allodynia evaluation (PTAE) of the right hind paw. The measure was obtained for the three groups (eight animals per group) in grams on days 3, 7, 14, and 21 following the intra-articular injection of monosodium iodoacetate (MIA). Intra-articular injection was performed on day 0 (2 mg of MIA for the MIA and rats injected with monosodium iodoacetate and punctual lidocaine (MIA-L) groups and 0.9 % NaCl for the sham group). The MIA-L group also received an intra-articular injection of lidocaine in the right knee on days 7, 14, and 21, at 25 minutes before the PTAE. *SD* standard deviation

#### Place escape/avoidance paradigm

**Fig. 5:**
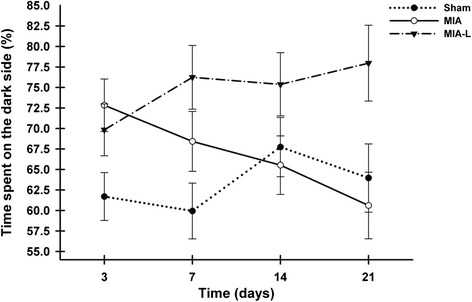
Place escape/avoidance paradigm (PEAP) evolution after osteoarthritis induction (least squares mean ± standard error of the mean). The percentage of time spent on the PEAP dark side was statistically higher in the lidocaine-treated rats with monosodium iodoacetate (*MIA-L*) when compared to the rats injected with monosodium iodoacetate (*MIA*) (*P* = 0.07) and the sham group (*P* = 0.01) group. Data presented here were collected for the whole period of assessment (20 minutes) at each day, but the observed between-group differences were the most obvious during the intermediate periods 2 (5–10 minutes) and 3 (10–15 minutes)

Between-group analysis confirmed a significant treatment effect of lidocaine, in which MIA-L was different from MIA (*P* = 0.07) and different from sham (*P* = 0.01) (Fig. 5). The group difference was particularly present for the two intermediate periods 2 and 3 of PEAP assessment previously observed as the most repeatable ones (see “Phase 1”).

#### Treadmill

There was close similarity in the type of performance on the treadmill in the MIA-L and sham groups, in which their TNTC remained comparable to baseline values. Inversely, the TNTC in the MIA group decreased from day 7 onward. However, the observed between-group difference was not significant (*P* = 0.14).

#### Neuropeptides

**Fig. 6:**
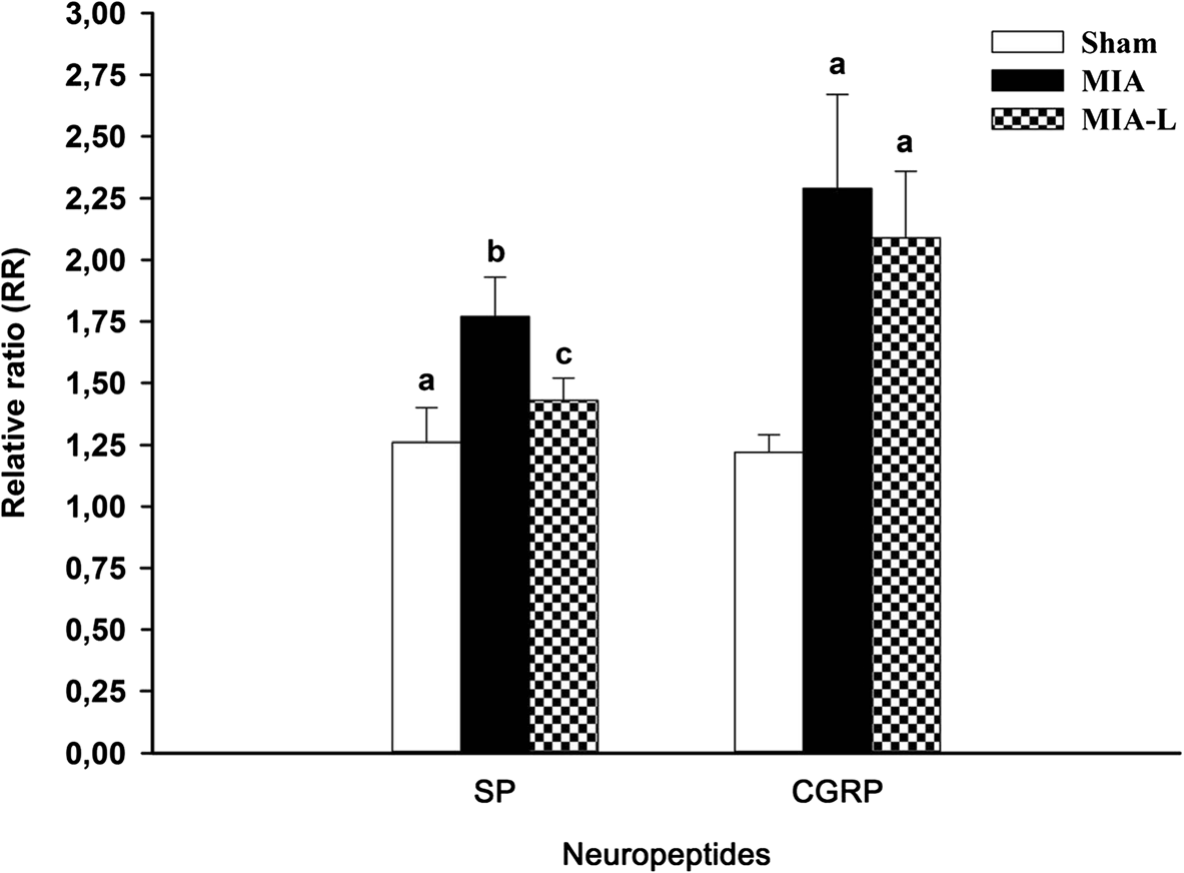
Spinal substance P (*SP*) and calcitonin gene-related peptide (*CGRP*) concentrations 21 days after monosodium iodoacetate (*MIA*) injection (relative ratio (*RR*) mean and SD). Mean RR spinal cord concentration was normalized to the naive group. An RR of 1 indicated the concentration of normal rats from the naive group. The RR for SP and CGRP were increased in all groups (including the sham group) but had a higher peak after MIA injection. Lidocaine treatment (*L*) induced a lesser liberation of SP and CGRP (albeit not statistically significant for the latter) in the spinal cord of the MIA-L group. ^a,b,c^Significant inter-group statistical differences (adjusted *P* value <0.10)

The mean relative ratio (RR) of neuropeptide concentrations of SP and CGRP 21 days after induction of OA are shown in Fig. 6. The absolute values of the concentration of neuropeptides have all been normalized to the function of the naive group values and are shown as the RR. Compared to the naive group (Table 5 and Fig. 6) with a RR of 1, the SP concentrations were significantly increased in the MIA model (adj-*P* = 0.016) with 2 mg of MIA (RR 1.77 ± 0.16) as in the lidocaine treatment group (MIA-L) (RR 1.43 ± 0.09). The level of this peptide was statistically higher in the MIA group (adj-*P* = 0.029) compared to the sham group injected with saline 0.9 % (RR 1.26 ± 0.14). However, both the sham and MIA-L groups have significantly lower SP concentrations when compared to the MIA group (adj-*P* = 0.029). The concentration of CGRP was significantly increased in both MIA models (RR 2.29 ± 0.39 and 2.09 ± 0.29 for MIA and MIA-L, respectively). The sham group (RR 1.22 ± 0.07) had an increase too, in comparison with the naive group (adj-*P* = 0.016). On the other hand, both MIA groups had a statistically significantly higher level of CGRP than the sham group (adj-*P* = 0.029). When compared to the MIA group, the MIA-L group had a statistically similar level of CGRP neuropeptide (adj-*P* = 0.200).

**Table 5:** Neuropeptides inter-group comparisons in the monosodium iodoacetate osteoarthritis rat model

Inter-group comparisons	SP	CGRP
Three groups vs. naive rats	0.016*	0.016*
MIA vs. sham	0.029*	0.029*
MIA-L vs. sham	0.057*	0.029*
MIA-L vs. MIA	0.029*	0.200

*MIA* monosodium iodoacetate, *MIA-L* rats injected with monosodium iodoacetate and treated with punctual lidocaine injection, *SP* substance P, *CGRP* calcitonin gene related-peptide. *Inter-group statistically significant difference (adjusted *P* value)

## Discussion

Rat models are common in OA research as they are easy to customize and are cost-effective [58]. The MIA model in particular can be standardized and is associated with rapidly developing well-characterized lesions [23, 30]. In an effort to improve the translation of preclinical OA research to the clinical field, we conducted a two-phase study, first, to determine the most reliable pain assessment method protocol, and second, to validate this protocol in the most common chemical model of OA in rats with concomitant changes in spinal neuropeptide concentrations.

Initially, the effect of the environment (inverted circadian cycle, activity level (treadmill exercise), and observer) was tested using well-known pain assessment tools. Prior studies have demonstrated an effect of the inverted circadian cycle on pain research protocols with rodents [59–61]. Our results did not suggest any impact of conducting the evaluation during daytime (more convenient for the investigator). Our group reported a significant reduction in variability of kinetics measures after exercise in cats [62] and dogs [63] with OA. The current study confirmed beneficial effects of exercise to reduce SWB (or other outcome) variability in the MIA rat model of OA. This study also qualified TNTC as a quantitative pain measure using spontaneous behavior. Importantly, the study tested reliability and validity of TNTC, and the potential impact on results obtained with other pain assessment methods, which may be used concurrently. Our results confirmed that a broad range of methods can be combined for pain assessment in the same animals while maintaining reliability and scientific validity.

Finally, as different observers can introduce some degree of bias in pain assessment outcomes, inter-rater reliability was tested by observers with different levels of expertise (one intermediate and one advanced). The methodology included in the current study was accessible to an observer with intermediate experience, as no significant difference was identified during analysis based on the level of experience. As a limitation, the number of observers was minimal, as both observers were women, and only objective assessment methods were selected for this study (limiting any bias related to subjective observation). Therefore, such a hypothesis (the potential influence of experience, and/or gender) would need to be tested further before making inferences from the results. This is particularly important, as recent work has established the influence of the observer’s gender in inducing stress-related analgesia in rodents [64]. Similarly, for limiting the influence of interferential factors in studying the effect of environment, only female rats were used. A possible gender effect would need to be tested in future experiments. Indeed, male rodents are recognized as more sensitive to olfactory exposure to males, including men, causing stress and related analgesia [64]. Moreover, sexual dimorphism [65] and hormonal influence [66, 67] have been observed in endogenous pain modulation mechanisms. Finally, women are more represented in the field of chronic pain [68]; however, many reasons have been explored to investigate this finding [69]. Also, for decades, males have been overrepresented in preclinical research. This situation can definitely lead to a certain bias [70]. All these previous studies justify our decision to use female rats.

As a recognized indicator of test-retest reliability [57], the ICC demonstrated that assessment of mechanical sensitivity using the Randall Selitto test presents poor repeatability, and this outcome cannot be recommended for a valid reflexive measure of pain. The phase 1 experiments demonstrated that SWB and tactile sensitivity can produce more repeatable data when animals (and the observer) are allowed to acclimate to the test device for at least one week. Similarly, there was a slight reduction in the variability of SWB when measured after treadmill exercise. However, the beneficial effects of exercise were not as significant in the chemically induced OA rat model as those observed in cats with naturally occurring OA [62, 71]. The PEAP, rotarod, and treadmill activity measured as TNTC appeared to be highly repeatable without requiring prolonged acclimatization. It must be noted that in both treadmill and PEAP, the intermediate periods 2 and 3 (i.e., 5–10 minutes, and 10–15 minutes, respectively) demonstrated the highest repeatability. These results also suggest that the treadmill and PEAP sessions are a little too long, so for future experimentation the session could be reduced in both cases to 15 minutes instead of 20 minutes. To our knowledge, this study is the first to evaluate the test-retest (repeatability) and inter-rater (reproducibility) reliability of a complete set of pain assessment methods in normal rodents.

As a measure of distribution dispersion that does not require similar units and therefore allows comparison of different variables, the CV of each pain assessment method was verified. At the first evaluation, we again observed the poor metrological property of mechanical sensitivity, presenting the highest inter-individual variability (around 70 % CV). Moreover, the different acclimatization protocols did not help to decrease this variability as the variation in CV from the last to the first evaluation was between 0.53 and 1.07. On the other side, our results support the importance of choosing the optimal acclimatization protocol for pain assessment in the rat model. To our knowledge, this study represents the first systematic evaluation of the effect on data variability of different acclimatization protocols, including four to eight assessments over a 2-week period, with a series of tests near or far from the others. The most reliable acclimatization protocol included five assessments with exposure to the testing methods every other day for the last week (days -14, -7, -5, -3, and -1), with baseline values acquired at day -1.

The second exploratory phase of our project evaluated the validity of the most promising pain assessment methods as determined during phase 1, when applied to the MIA model of OA in rats. Briefly, SWB, PTAE, PEAP, and TNTC detected pain-related changes following OA induction with an intra-articular MIA injection and were validated by increased release of spinal neuropeptides such as SP and CGRP. However, the rotarod assessment, as used in our experimental conditions, was not sensitive to induction of OA pain. Moreover, the PTAE and PEAP methods demonstrated that the sham injection of 0.9 % NaCl was not totally neutral, which was confirmed by the augmentation of spinal liberation of SP (26 times higher) and CGRP (22 times higher) in the sham group. Interestingly, PTAE and PEAP also confirmed the analgesic effect of intra-articular lidocaine injection by the downregulation of spinal neuropeptides, whereas TNTC and SWB did not detect the expected analgesic effect. These results suggest that SWB detects more biomechanical alterations of the joint than ongoing pain and consequently, could be a sensitive method to detect knee joint dysfunction. Of the four pain assessment methods evaluated for concurrent validity, only PEAP detected a treatment effect of lidocaine with a significant difference between the MIA-L group and both the sham and MIA groups. Interestingly, body weight was not affected, either by possible manipulation-related stress in phase 1, or by MIA induction of pain in phase 2. This confirms the possible lack of sensitivity of different endpoints used in research, such as feeding, drinking, etc., for determining quality of life.

The enhanced escape/avoidance behavior and lower PWT were previously demonstrated in both neuropathic and inflammatory pain models [33, 41, 47, 72, 73]. The higher sensitivity of PEAP compared to PTAE in detecting the efficacy of pain relief was also demonstrated in other studies [33].

It would be logical to consider that animals would allocate roughly a similar amount of time in both environments if they do not show natural preference or aversion to one of the two environments in the PEAP testing. This study clearly establishes a strong preference of the rat, a nocturnal animal, to the dark side of the test apparatus. The acclimatization of rats to the test apparatus is fast, the establishment of a baseline is preferable, and both intermediate periods 2 and 3 are highly repeatable. Moreover, PEAP assessment includes both classical (Pavlovian) and operant conditioning in the process of training [42]. When compared with PEAP, PTAE required a longer acclimatization period for both the animal and the observer (at least one week as demonstrated in this study). However, assessing the escape/avoidance behavior using the PEAP required a much longer evaluation time (with 20 to 30 minutes required for each animal), being too labor-intensive to the experimenters, while no automated apparatus is commercially available for this test.

Interestingly, the intra-articular lidocaine injection affected both PTAE and PEAP, and to a lesser degree TNTC, but did not alter changes in weight bearing on the RHP. The lack of effect of lidocaine on SWB could be related to reduced pain in the absence of movement in this model. In a recent study [25], intra-articular lidocaine (200 μL) was efficient to reduce the shift in weight bearing at day 14 post-MIA injection, but only for the highest dose of MIA (4.8 mg). The lower MIA dose and volume in our study (2 mg and 50 μL) combined with the lower sensitivity of SWB may be responsible for the lack of effect with this method, while PEAP and PTAE accurately captured the expected pharmacological effects of lidocaine. Intra-articular lidocaine was chosen for the analgesic test in this study, because of the apparently controversial results obtained in conditioning procedures with non-steroidal anti-inflammatory and opioid drugs (for review, see [42]).

These findings may be useful when designing studies of the efficacy of analgesia using the MIA-induced OA rat model. Moreover, there is also some evidence that the combination of the quick-acting effect of lidocaine (reaching a peak effect at 10 minutes after the intra-articular injection on a CatWalk) [74] and the necessary time to induce a change in the distribution of gait in supraspinal locomotor areas in patients with OA [75], seems to explain the lack of detection of lidocaine analgesia by SWB. Previous studies combined with our results, provide evidence for future use of a continuous infusion of lidocaine to obtain a more sustained analgesic effect, attaining higher lidocaine synovial levels for a prolonged time period.

The intra-articular injection of saline (sham group) generated some hyperalgesia or allodynia, as assessed by PTAE and reflected by the observed change in the operant testing. This is supported by the recent finding of some increased NF-kB activity on days 3 and 7 measured by in vivo luminescent imaging in a transgenic mouse model receiving an intra-articular injection of saline [76]. Moreover, in the MIA mouse model tested in the same study, temporal kinetics of NF-kB activity were strongly correlated with mechanical allodynia (PTAE) and serum interleukin (IL)-6 levels in the inflammatory phase (day 3) of this model, while serum IL-1β was strongly correlated with pain sensitivity in the chronic pain phase (up to day 28) [76]. An increase in the intra-articular pressure and possible injection-related inflammation are proposed to explain this finding. Based on these results, a neutral control group (without intra-articular injection) may be valuable in future experiments.

The MIA model is recognized as valuable in OA research for its ability to detect analgesic effects of different drugs and compounds. The initial inflammatory phase of this model allows the evaluation of various non-steroidal anti-inflammatory drugs and cyclo-oxygenase inhibitors [14, 24, 27, 77]. Moreover, the efficacy of morphine, gabapentin, pregabalin, and the transient receptor potential vanilloid receptor antagonist was successfully demonstrated in this model [15–17, 24, 77]. Moreover, some studies showed that MIA-induced OA leads to an increase in the neuron firing rate and a reduced activation threshold of the afferent nerve fibers [78], which consequently leads to sensitization of spinal neurons in the dorsal horn [79]. In this study, spinal cord neuropeptide quantification suggests and supports development of central sensitization in this model. Indeed, our study confirms an increase in spinal biomarkers of SP and CGRP, as previously observed in the MIA model [51].

The upregulation of spinal neuropeptides observed in this study suggests activation of the peptidergic afferent C-fibers, resulting in central sensitization. It has been well-demonstrated [80], by relative increasing expression of target gene mRNA like pro-inflammatory cytokines (IL-1 and tumor necrosis factor) and pain mediators (CGRP, SP, neuropeptide Y, and galanin), that MIA-induced joint degeneration in rats generates an animal model suitable for mechanistic and pharmacologic studies on nociceptive pain pathways caused by OA. Altogether, this provides further key in vivo evidence that OA pain could be caused by central sensitization through communication between peripheral OA nociceptors and the central sensory system [81, 82]. Despite the fact that SWB did not detect lidocaine treatment on day 21, or asymmetry of weight distribution, our study clearly demonstrated that lidocaine analgesic effects noted by PEAP were translated by concomitant significant downregulation of spinal SP, which was 34 times lower, and of CGRP, which was 20 times lower on day 21.

These results mimic similar therapeutic effects on behavior and SP and CGRP spinal cord expression of intra-articular resiniferatoxin [83] and proteasome inhibitor MG132 [84] in the MIA OA pain model in rats. Unfortunately, we observed that the MIA model caused temporary changes of short duration (return to baseline values at day 21 post injection) and relies on a disease mechanism (chemical inhibition of glyceraldehyde-3-phosphate dehydrogenase activity in chondrocytes, resulting in cell death following the disruption of its cellular glycolysis process [15, 16, 18]) that differs from human natural OA, which could limit the predictability of the therapeutic effect of analgesic and disease-modifying agents. Finally, higher levels of spinal neuropeptides at sacrifice clearly confirms that our model caused some long-term pain or OA damage.

## Conclusion

Pain assessment methods used with the MIA model should be selected and scheduled appropriately. In this study only mechanical sensitivity had poor metrological properties, but SWB, the operant PEAP testing, tactile sensitivity, rotarod, and treadmill (TNTC) were repeatable under different environmental conditions. The rotarod test did not achieve sufficient sensitivity to detect OA pain induced by MIA injection in rats and may not be included in future studies. For detecting the analgesic effect of local administration of lidocaine, the pain assessment method that demonstrated the best results was the operant testing, which had the greatest sensitivity, followed by PTAE, whereas SWB had some limitation in sensitivity. Spinal neuropeptide quantification at the end of the experiment has allowed us to validate the effect of positive lidocaine treatment in a more objective manner, as MIA can induce pain. However, the main limitation of this study was the small sample size. Furthermore, it was possible to increase the validity and reliability of pain assessment methods with an optimal acclimatization protocol (five assessments over 2 weeks). In addition, the sham intra-articular saline injection was not totally neutral, particularly with more sensitive methods such as PEAP, PTAE, and this was confirmed by the release of spinal neuropeptides. We therefore recommend the addition of a naive control group (without intra-articular injection). Moreover, increased neuropeptide levels obviously support the central sensitization observed in the MIA rat model. The present results highlight potential for these neuro-mediators as pharmacological biomarkers for analgesic testing in association with sensitive functional assessment methods.

## Abbreviations

Adj-P, adjusted *p* value; BW, body weight; CGRP, calcitonin gene-related peptide; CV, coefficient of variation; D, day; HPLC-MS/MS, tandem mass spectrometry coupled to high-performance liquid chromatography; ICC, intraclass correlation coefficient; IL, interleukin; L, lidocaine; LHP, left hind paw; LS-Mean, least square means; MIA, monosodium iodoacetate; MRM, multiple reactions monitoring; MS, mechanical sensitivity; n, number of animals; OA, osteoarthritis; *P*, probability; PEAP, place escape/avoidance paradigm; PTAE, punctate tactile allodynia evaluation; PWT, paw withdrawal threshold; RHP, right hind paw; RPM, revolutions per minute; RR, relative ratio; SD, standard deviation; SEM, standard error of the mean; SP, substance P; SWB, static weight bearing; TNTC, treadmill number of total crossings; TS, tactile sensitivity; V/V, volume/volume
